# A Systematic Review and Meta-Analysis on the Longitudinal Effects of Unilateral Knee Extension Exercise on Muscle Strength

**DOI:** 10.3389/fspor.2020.518148

**Published:** 2020-11-16

**Authors:** Ekin Altan, Svenja Seide, Ismail Bayram, Leonardo Gizzi, Hayri Ertan, Oliver Röhrle

**Affiliations:** ^1^Department of Continuum Biomechanics and Mechanobiology, Institute for Modeling and Simulation of Biomechanical Systems, University of Stuttgart, Stuttgart, Germany; ^2^Institute of Medical Biometry and Informatics, University of Heidelberg, Heidelberg, Germany; ^3^Department of Coach Training in Sports, Faculty of Sport Sciences, Eskisehir Technical University, Eskisehir, Turkey; ^4^Coaching Education Department, Faculty of Sport Sciences, Eskisehir Technical University, Eskisehir, Turkey

**Keywords:** systematic review, knee extension, unilateral isometric exercise, healthy subjects, longitudinal model-based meta-analysis

## Abstract

The aim of the study was to investigate the time-dependent increase in the knee extensors' isometric strength as a response to voluntary, unilateral, isometric knee extension exercise (UIKEE). To do so, a systematic review was carried out to obtain data for a Bayesian longitudinal model-based meta-analysis (BLMBMA). For the systematic review, PubMed, Web of Science, SCOPUS, Chochrane Library were used as databases. The systematic review included only studies that reported on healthy, young individuals performing UIKEE. Studies utilizing a bilateral training protocol were excluded as the focus of this review lied on unilateral training. Out of the 3,870 studies, which were reviewed, 20 studies fulfilled the selected inclusion criteria. These 20 studies were included in the BLMBMA to investigate the time-dependent effects of UIKEE. If compared to the baseline strength of the trained limb, these data reveal that UKIEE can increase the isometric strength by up to 46%. A meta-analysis based on the last time-point of each available study was employed to support further investigations into UIKEE-induced strength increase. A sensitivity analysis showed that intensity of training (%MVC), fraction of male subjects and the average age of the subject had no significant influence on the strength gain. Convergence of BLMBMA revealed that the peak strength increase is reached after ~4 weeks of UIKEE training.

## 1. Introduction

Understanding muscle growth and adaptation in response to exercise is the basis for a healthier and a more active lifestyle (Tesch, 1988; Egan and Zierath, 2013). Training our muscular system in an effective and controlled way may lead to increased mobility (Daley and Spinks, 2000), fosters faster recovery from injuries or surgical interventions through specialized, and thus, more efficient and subject-specific training/rehabilitation programs (Warburton et al., 2005; Plüss et al., 2018), and potentially leads to performance enhancement in (professional) athletes (Myer et al., 2005). Elderlies, for example, can greatly benefit from an increase in strength (Harridge et al., 1999), as it reduces the risks of falling and their psychological consequences, promotes healthy aging and fosters independent living. Since muscle growth and adaptation is a very active field of (experimental) research, there exists a large body of literature, including several detailed systematic reviews on dynamic exercise modes (e.g., Roig et al., 2008; Krieger, 2010; Schoenfeld et al., 2016, 2017). Despite the large body of literature, the mechanisms of training-induced muscle adaptation are generally still not that well-understood (Timmons, 2011). By restricting oneself to a particular and “simple” type of exercise, such as, for example, isometric training and a specific muscle, there is hope that one can reveal by means of systematic investigations the complex mechanisms leading to muscle growth or adaptation.

Isometric training is a form of exercise that consists of muscle contractions performed without macroscopic changes to the position, i.e., without lengthening or shortening of the muscle belly (Enoka, 1988). If new insights can be gained from isometric training studies, specific training plans can be developed that can easily be employed in a home setting, since it requires little to no equipment to be performed, and is relatively easy to describe and to assess (Oranchuk et al., 2019). Further, it leads to an increase in muscle force with exerting minimal stress to the structures surrounding the involved muscles. This makes the experimental outcome less susceptible to inter-muscle force transmission and especially suitable for post-surgery rehabilitation (Jaramillo et al., 1994). Moreover, the controlled environment and its intrinsic reduction in the risk of training-related injuries, also make isometric training highly suitable for elderly populations.

Moreover, studies, in which the training is isometric and unilateral, provide a natural way to study the influence of training by comparing the trained limb with the contralateral limb. There exists a number of studies on unilateral training of the upper extremities (e.g., Rasch and Morehouse, 1957; Meyers, 1967; Coleman, 1969; Ikai and Fukunaga, 1970; Knapik et al., 1983; McDonagh et al., 1983; Davies et al., 1988; Thepaut-Mathieu et al., 1988; Kitai and Sale, 1989; Herbert et al., 1998; Macaluso et al., 2000; Ebersole et al., 2002; Kanehisa et al., 2002; Colson et al., 2009; Lee et al., 2009; Driss et al., 2014) and the lower ones (e.g., Young et al., 1985; Alway et al., 1989; Behm and Sale, 1993; Burgess et al., 2007; Del Balso and Cafarelli, 2007) as well as the hand (e.g., Darcus and Salter, 1955; Duchateau and Hainaut, 1984; Cannon and Cafarelli, 1987; Davies et al., 1988; Yue and Cole, 1992; Patten et al., 2001; Carroll et al., 2002; Manca et al., 2016). Most unilateral isometric knee extension (UIKEE) studies focus on neuromechanical changes by analysing EMG data (e.g., Lewis et al., 1984; Carolan and Cafarelli, 1992; Garfinkel and Cafarelli, 1992; Bandy and Hanten, 1993; Weir et al., 1994, 1995; Rich and Cafarelli, 2000; Balshaw et al., 2016, 2017; Ema et al., 2017), morphological changes through monitoring muscle mass/cross-sectional area/volume (e.g., Lewis et al., 1984; Jones and Rutherford, 1987; Garfinkel and Cafarelli, 1992; Kubo et al., 2001; Balshaw et al., 2016, 2017), comparison of the training effect of UIKEE with non-isometric modes of training (e.g., Bonde Petersen, 1960; Parker, 1985; Rutherford and Jones, 1986; Jones and Rutherford, 1987; Folland et al., 2000, 2005; Lee et al., 2018), changes in co-activation of the synergistic muscles (e.g., Carolan and Cafarelli, 1992; Tillin et al., 2011), cross-education (e.g., Lewis et al., 1984; Weir et al., 1995), comparison of externally stimulated and voluntary exercises on the training outcome (e.g., Mohr et al., 1985; Hartsell, 1986; Kubiak et al., 1987; Baskan et al., 2011), specificity of the joint angle and it's influence at the trained as well as non-trained angles (e.g., Bandy and Hanten, 1993; Weir et al., 1994, 1995), and metabolic changes (e.g., Grimby et al., 1973; Komi et al., 1978; Lewis et al., 1984).

Besides these studies, researchers have investigated the implications of isometric training on the management of blood pressure (e.g., Owen et al., 2010; Carlson et al., 2014; Inder et al., 2016). Although Munn et al. (2004) and Bohm et al. (2015) did not merely include isometric exercise in their systematic reviews, they included isometric exercise studies on various muscles/muscle groups as they concentrated on the strength gain in the contralateral limb and the impact of the exercise on tendon stiffness, respectively. By pooling studies on the training of large muscles, Oranchuk et al. (2019) focused on the longitudinal adaptation due to isometric exercise, but did not distinguish between uni-/bilateral training. None of these studies focuses on the strength gain in the trained leg due to unilateral isometric exercise, which is the focus of our study.

Since strength increase, i.e., the training outcome, is known to significantly depend on duration of the trained period (Powers and Howley, 2007), longitudinal studies are essential. Longitudinal studies quantifying the improvement in performance, however, require detailed knowledge about the training outcome, ideally after each training session. Due to the absence of an universal consensus on training modality, it is not possible to use existing data in a straight forward manner to conduct a longitudinal analysis.

To overcome this limitation, we employ a model-based meta-analysis combining longitudinal models with meta-analytic methods to synthesize results from different primary trials (Pedder et al., 2019). Generally speaking, model-based meta-analysis is based on the Bayesian paradigm that allows to combine statistical modeling and meta-analysis within one model. Specifically herein, we use a Bayesian, longitudinal model-based meta-analysis (BLMBMA) as proposed in Boucher and Bennetts (2018) to analyse data originating from different studies. This method allows to fit a longitudinal model to the data, hence, the evolution of the training effect can be observed. Furthermore, model-based meta-analysis reduces between-trial heterogeneity and leads to an increased precision in the estimation of the overall training outcome. Therefore, the aim of the study was to investigate the time-dependent increase in the knee extensors isometric strength as a response to UIKEE through a systematic review and BLMBMA.

## 2. Methods

### 2.1. Study Selection

Articles in the databases PubMed, Web of Science, SCOPUS, and Chochrane Library were systematically reviewed. The search was performed on the title and abstract (see section 1 in Supplementary Material for the full search strategy). The last search took place in May 2019. The database search, screening, and data extraction were performed by authors EA and IB. References of the relevant articles as well as reviews on the topic of resistance training were also investigated manually by the authors EA and IB. The PRISMA Statement (Moher et al., 2009) was followed.

### 2.2. Inclusion Criteria

Only studies on voluntary UIKEE performed by young, healthy subjects were included within this review. Further, the search was restricted to articles in English and published after 1960.

As the neuromuscular system is thought to adapt itself to perform the (specific) training movement in an optimized manner (Morrissey et al., 1995; Powers and Howley, 2007), the principle of exercise-specificity states that the highest effect of exercise is assumed when the training (e.g., training angle, mode) and the measurement of the training outcome overlap. Adhering to the principle of exercise-specificity, i.e., strength gain coincides with training (e.g., training angle, mode), a study was included only if it reported the isometric strength at pre- and post-training stages in terms of the maximum voluntary contraction/torque (MVC/MVT) of the trained limb.

In addition, if a study used different subject groups for different training modes, and one of these training modes was UIKEE, only data reported from the UIKEE group was taken into consideration.

### 2.3. Exclusion Criteria

Studies following a training plan according to the principle of “training to exhaustion” were excluded as one cannot quantify in such studies the number of contractions per training session. Moreover, training with <50% MVC was deemed to be inadequate to impose strength improvement in the trained muscle (Munn et al., 2004). Hence, studies reporting training intensity lower than 50% MVC were excluded. The same applies to studies accompanying training with electrical stimulation (during the warm-up and/or training). They were disregarded to minimize the training protocol heterogeneity.

It is known that training in one limb may cause strength improvement on the contralateral limb (see e.g., Carolan and Cafarelli, 1992; Weir et al., 1994; Griffin and Cafarelli, 2005), which is known as cross-education (Enoka, 1988, 1997). If a study reports on training protocols that differed from side to side, e.g., different angle, eccentric/concentric training, then the study was also excluded from the review. Furthermore, by focusing on unilateral exercise, studies reporting on some acute effects like the bilateral deficit phenomenon, which occurs in concurrent contraction of homologous muscles and yields reduced strength gain on both limbs, were disregarded (Howard and Enoka, 1991). Studies merely utilizing a bilateral training protocol or training the limbs in alternating manner were also excluded.

When progressive training programs are planned for elderly populations, the baseline fitness and potential pre-existing medical conditions need to be taken into consideration (Greig et al., 1994; Macaluso and De Vito, 2004). Although it is known that elderly subjects also immensely benefit from resistance training, strength gains are dependent on the baseline fitness levels of the subject population (Kraemer and Ratamess, 2004). The fact that the baseline fitness levels are typically lower for elderly subjects than for younger ones (Lindle et al., 1997) leads to increased data heterogeneity if such data are combined. Therefore, to keep the potential heterogeneity stemming from the demographics and health state of the subjects to a minimum, studies on elderly population, as well as, studies on young subject with pre-existing medical conditions or lower limb injuries were excluded.

### 2.4. Data Collection and Extraction

One of the most prominent functional adaptation due to resistance training is improved muscle strength, which also indicates how well the trained muscle adapts to the training regimen (Kraemer and Spiering, 2006). Therefore, the isometric strength of the trained leg is chosen as the main summary measure. The secondary summary measures were chosen from quantities which are related to the causes of the strength improvement, namely the neuromechanical and morphological changes of the trained muscle.

EMG measurements, for example, are used to assess changes in the neuromuscular system (Aagaard, 2003; Griffin and Cafarelli, 2005; Enoka, 2008). They can be reported in terms of integrated EMG or normalized root mean square (RMS). Whereas, measurements on the muscle mass, cross-sectional area (CSA) or volume describe changes to the morphological properties. Positive changes in the morphological properties point to an increase in the amount of contractile proteins, known as hypertrophy (Enoka, 2008). EMG and morphological data were considered as secondary summary measures.

Demographical information related to the participants (age, weight, height, sex, history of physical activity) and information regarding the training variables employed in the studies were also extracted. The extracted training variables were: exercise intensity (in terms of % MVC), knee angle, number of contractions per set, rest between contractions, number of sets per training, rest between sets, contraction duration, number of training sessions per week and the weeks trained.

Unfortunately, the identified trials differed widely in design and not all of them were controlled trials. Furthermore, some of the studies with a controlled design relied on internal controls (the trained vs. the untrained leg), while others reported results from external control groups (trained vs. untrained participants). We were therefore unable to obtain a sufficiently large data set for either of the types of controls. Thus, our main analysis relied on uncontrolled data, stemming from trained legs, and analyses using the two types of control were added as sensitivity analysis.

When multiple UIKEE exercise protocols (e.g., training at different knee angles, intensities) were used for different subject groups within one study, the data on different exercise protocols were treated as separate data sets (see e.g., Bonde Petersen, 1960; Szeto et al., 1989; Bandy and Hanten, 1993). The reason for this is related to the principle of exercise-type specificity, which asserts that differences in the training protocols should be taken into account when the outcome of the training is evaluated (Morrissey et al., 1995).

The treatment effect of the summary measures were computed based on the change in the summary measure with respect to its baseline value, i.e., value measured in the pre-exercise state. Muscle strength can be measured through MVT, MVC, or weights, whereas, volumetric data can be reported through muscle mass, CSA or volume. Computing the relative change of these measures and using this as the treatment effect allows to evaluate the observed changes using a common unit.

The treatment effect in week X relative to its baseline was computed as:

(1) $$\begin{array}{l} {\text{TE}}_{\text{X}}=\left(\frac{\Delta_{\text{X}}}{{\text{Y}}_{\text{0}}}\right)100=\frac{{\text{Y}}_{\text{X}}-{\text{Y}}_{\text{0}}}{{\text{Y}}_{\text{0}}}*100, \end{array}$$

where TE denotes the treatment effect, X denotes the week number for which the data was reported, i.e., *X* = 0 refers to pre-exercise, and Y_(·)_ stands for summary measure at week (·). The change in the summary measure is denoted by Δ_X_.

The variance of TE_X_ is needed for the meta-analysis. It is not possible to compute this quantity using conventional ways, since Δ_X_ and Y_0_ in Equation (1) were both variables. The variance is thus approximated by means of a Taylor expansion. Following Munn et al. (2004),

(2) $$\begin{array}{l} \text{var}\left({\text{TE}}_{\text{X}}\right)=\text{var}\left(\frac{\Delta_{\text{X}}}{{\text{Y}}_{\text{0}}}\right)=\frac{\text{var}\left(\Delta_{\text{X}}\right)}{{\bar{{\text{Y}}_{\text{0}}}}^{2}}+\frac{\text{var}\left({\text{Y}}_{\text{0}}\right){\bar{\Delta_{\text{X}}}}^{2}}{{\bar{{\text{Y}}_{\text{0}}}}^{4}}\\ \;-\frac{2\bar{\Delta_{\text{X}}}\text{cov}\left(\Delta_{\text{X}},{\text{Y}}_{\text{0}}\right)}{{\bar{{\text{Y}}_{\text{0}}}}^{3}}, \end{array}$$

where var(·), cov(·), and $\bar{\left(\cdot \right)}$ denote the variance, covariance, and the mean of quantity (·), respectively.

The variance of Δ_X_ is denoted by var(Δ_X_) and is calculated as

(3) $$\begin{array}{l} \text{var}\left(\Delta_{\text{X}}\right)=\text{var}\left({\text{Y}}_{\text{X}}-{\text{Y}}_{0}\right)=\text{var}\left({\text{Y}}_{\text{X}}\right)+\text{var}\left({\text{Y}}_{0}\right)\\ \;-2*\text{cov}\left({\text{Y}}_{\text{X}},{\text{Y}}_{0}\right). \end{array}$$

Following (Munn et al., 2004), the correlation between (Y_X_, Y_0_) is taken as 0.5 and is used to for both cov(Y_X_, Y_0_) and cov(Δ_X_, Y_0_).

### 2.5. Synthesis of Results

**Figure 1:**
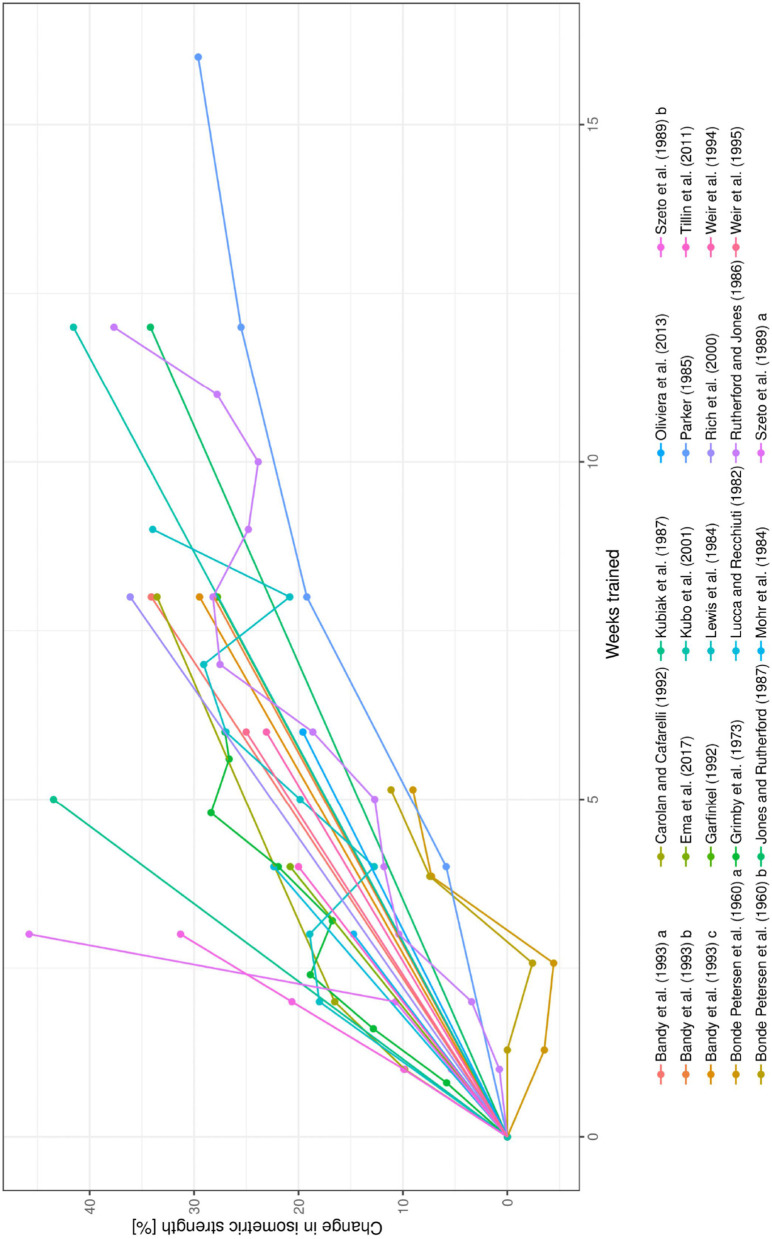
Spaghetti plot of the data on change in isometric strength over time in weeks for all studies included in the systematic review.

**Figure 2:**
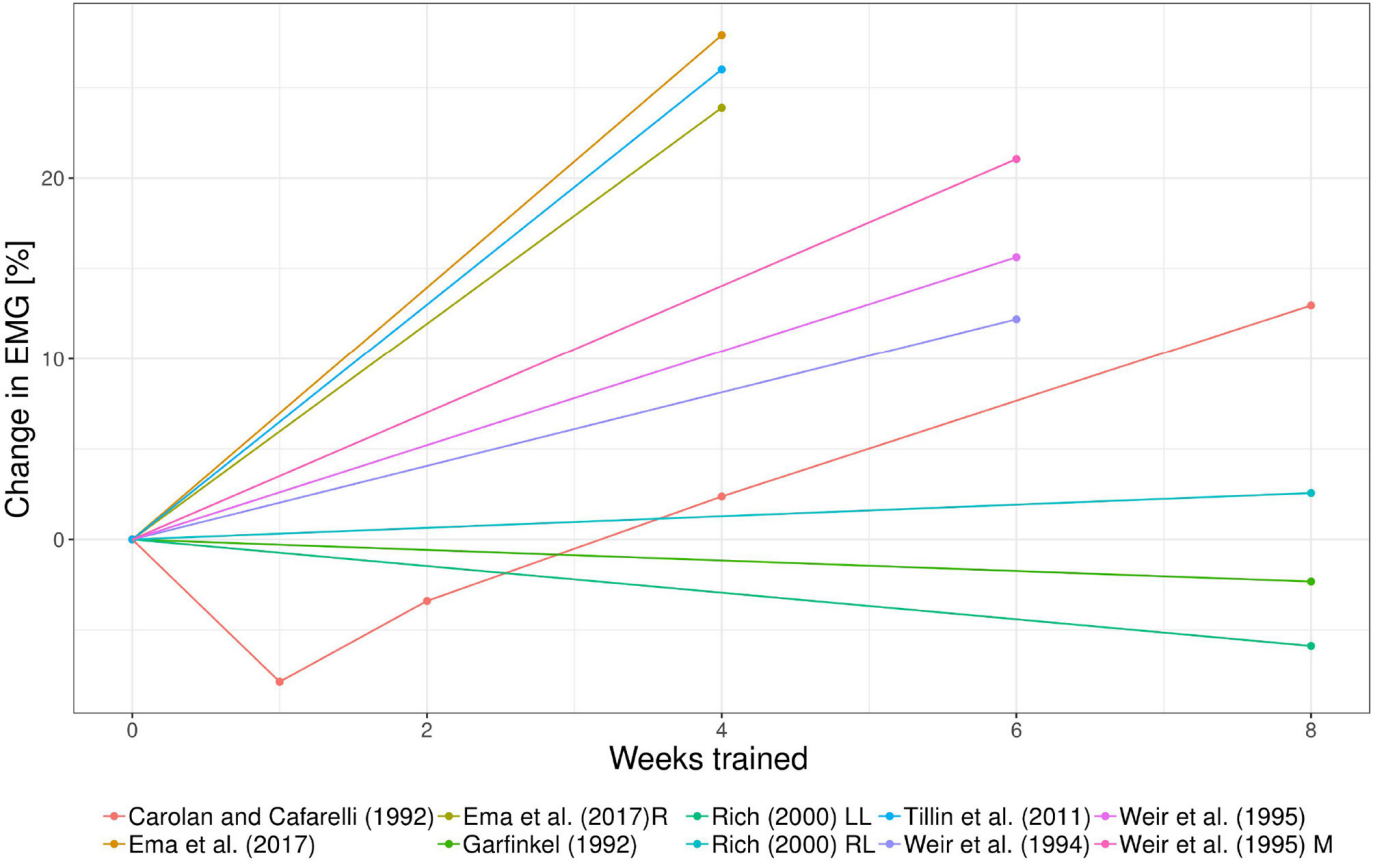
Spaghetti plot of the data on EMG over time in weeks obtained from Carolan and Cafarelli (1992), Garfinkel and Cafarelli (1992), Weir et al. (1994, 1995), Rich and Cafarelli (2000), Tillin et al. (2011), and Ema et al. (2017). Here R, rectus femoris; LL, left limb; RL, right limb; and M, vastus medialis. Where not specified, data were obtained from vastus lateralis muscle.

First, longitudinal information for the increase in strength and change in EMG within each study was evaluated descriptively to observe whether the data follow a trend. This was done using spaghetti-graphs over the respective time period (see Figures 1, 2). Each data set is illustrated as separate lines. The treatment effect at a given time point is expected to depend on weeks trained and exercise intensity.

BLMBMA makes it possible to model the time-course of the evolution of the treatment effect by using data reported at multiple end points. Furthermore, it is not constricted to a single time point like a landmark meta-analysis (Boucher and Bennetts, 2016). The time-dependent evolution of the treatment effects were modeled here using BLMBMA.

The Emax model is fitted to the data as proposed in Boucher and Bennetts (2016). This model captures the initial fast increase in performance improvement followed by a plateau, which is the case for subject with no prior strength training background (Powers and Howley, 2007). The Emax model has the following form:

(4) $$\begin{array}{l} \text{Effect}=E_{0}+\frac{E_{\text{max}}C}{C_{50}+C}, \end{array}$$

where *E*_0_ and *E*_max_ denote the baseline and the maximum effect of the drug, respectively, *C* is the drug concentration and *C*_50_ is the drug concentration at which 50% of the maximum effect is observed. The concentration-related parameters in Equation (4) were replaced with the weeks trained and the effect related parameters [*E*_(·)_] that describe the treatment effect, i.e., the isometric strength, CSA or EMG.

To apply BLMBMA, first, admissible sets for the values of the model parameters *E*_max_ and *C*_50_ were created. Such sets were known as prior sets in Bayesian statistics and they describe the value a given parameter may take, without considering any quantitative evidence for both parameters. Prior sets from a normal distribution with a mean of 0 and standard deviation 10,000, i.e., N(0,10,000), were created. Such priors are known as weakly informative as they follow a normal distribution with a large variance, resulting in a flat distribution that reflects the lack of previous knowledge on that parameter.

The posterior density of the parameters describes the values, which the parameters can take after the data at hand is taken into consideration. To obtain the posterior density, Markov chain Monte Carlo method was used. Three Markov chains with 10,000 repetitions each and a thinning factor of 10 were used to ensure convergence of the posteriors after a burn-in of 2,000 observations. In addition to thinning, we discarded the burn-in samples and used multiple chains to account for an unlucky choice of initial values in this numerical process. In each trial, the baseline parameter, *E*_0_, was set to 0. This is to account for the baseline adjustment in the individual studies.

Heterogeneity between studies was reflected on the *E*_max_ parameter by including a normally distributed random-effect on this parameter. This allows the *E*_max_ to vary between trials. Convergence of the estimation was assessed by means of Gelman-Rubin diagnostics, which gets close to 1 if convergence is reached (Gelman and Rubin, 1992). This estimation was performed in R (R Core Team, 2018) and jags (Plummer, 2003) using the extensions rjags (Plummer, 2018), tidyverse (Wickham et al., 2019), ggplot2 (Wickham, 2016), and meta (Balduzzi et al., 2019).

To illustrate the differences between individual studies, the increase in strength at the last available point in time per study was shown in a forest plot. Note that the forest plots were generated with meta (Balduzzi et al., 2019) in the software R (R Core Team, 2018). The plot was stratified by training duration and ordered by training intensity within similar training durations. This descriptive analysis was completed by a using the latest available time-point in each trial.

To evaluate the combined effect of this landmark analysis, a pairwise random-effects meta-analysis was carried out based on the last available observation of each study. The *I*^2^ statistic was used to evaluate the amount of heterogeneity between the pooled studies in all cases. Potential study-level covariates were included in univariate and multiple meta-regression in a further step of the landmark analysis. This was done to explain the variation between studies.

### 2.6. Risk of Bias in Individual Studies

The bias types outlined in the Cochrane Handbook for Systematic Reviews of Interventions version 5.2.0 were followed to assess the risk of bias (RoB) within this study (Higgins and Green, 2008). The “Cochrane risk-of-bias tool for randomized trials (RoB 2)” (Sterne et al., 2019) was used for this purpose. The summary of the assessment is provided in Supplementary Figure 1. Since there is no tool to assess studies that do not employ a control group, we excluded studies that do not report data on an independent control group or the untrained leg.

Authors EA and IB evaluated RoB independently. The results were compared until a consensus was reached. The RoB were assessed as follows:

- Bias due to the randomization process: This bias was assessed according to whether or not the subjects were assigned to the control or trained group according to random allocations. Baseline demographics as well as fitness level of the study participants were also assessed in this section.
- Bias due to deviations from intended interventions: This bias was assessed whether factors that might influence the strength improvement, namely the food intake (Mithal et al., 2013), daily activity during the exercising period (Caspersen et al., 1985), previous training experience (Ritti-Dias et al., 2011), presence of verbal encouragement or biofeedback (Lucca and Recchiuti, 1983), and manipulations to the muscle, e.g., biopsy (Costill et al., 1988), were kept constant during the training period.
- Bias due to missing outcome data: This bias type was assessed whether the number of subjects that withdrew from the study was statistically significant.
- Bias due to presentation of the measured results: This bias was assessed if results of the intermediary as well as pre-and post-training tests were reported in a tabular form or within a figure.
- Bias due to the selection of the reported result: Studies included in the review were assessed whether they report outcomes of the training effect they investigate and which were described in the methodology.

## 3. Results

### 3.1. Summary of Evidence

**Figure 3:**
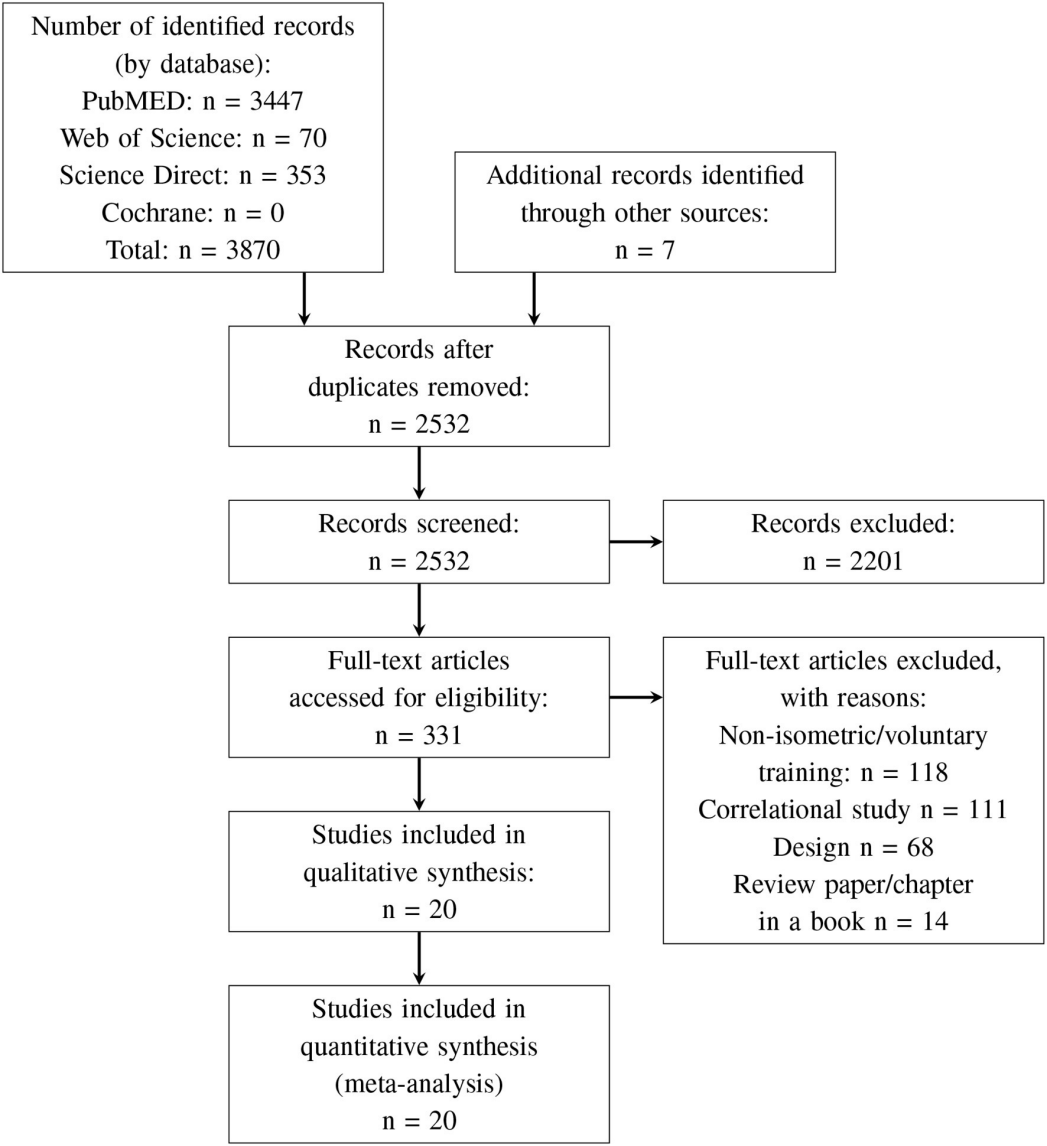
PRISMA 2009 flow diagram.

The database search was conducted in May 2019. The search yielded 20 studies fulfilling the inclusion criteria (see Figure 3).

**Figure 4:**
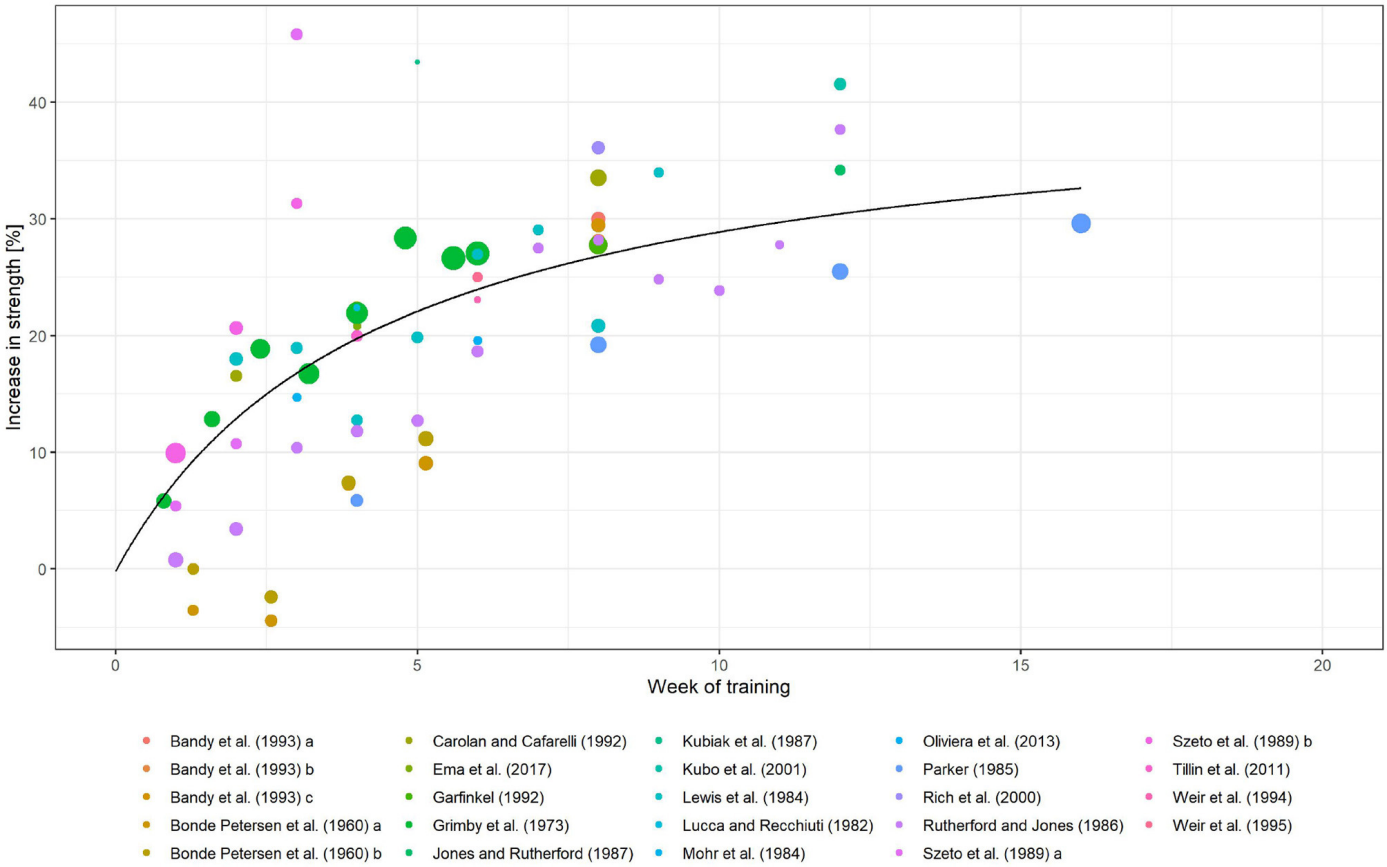
Fitted Emax model (black line) for the relative change in isometric strength over weeks trained together with data points obtained from multiple studies (colored dots). Note that the size of the colored dots are proportional to the subject size of a given study.

These studies contained 65 data points. The main summary measure (isometric strength) was computed based on these data points. Except for Bonde Petersen (1960), all studies reported a positive change with respect to the isometric strength of the trained leg. The change in isometric strength ranged from −4 to 46% (see Figure 4).

**Figure 5:**
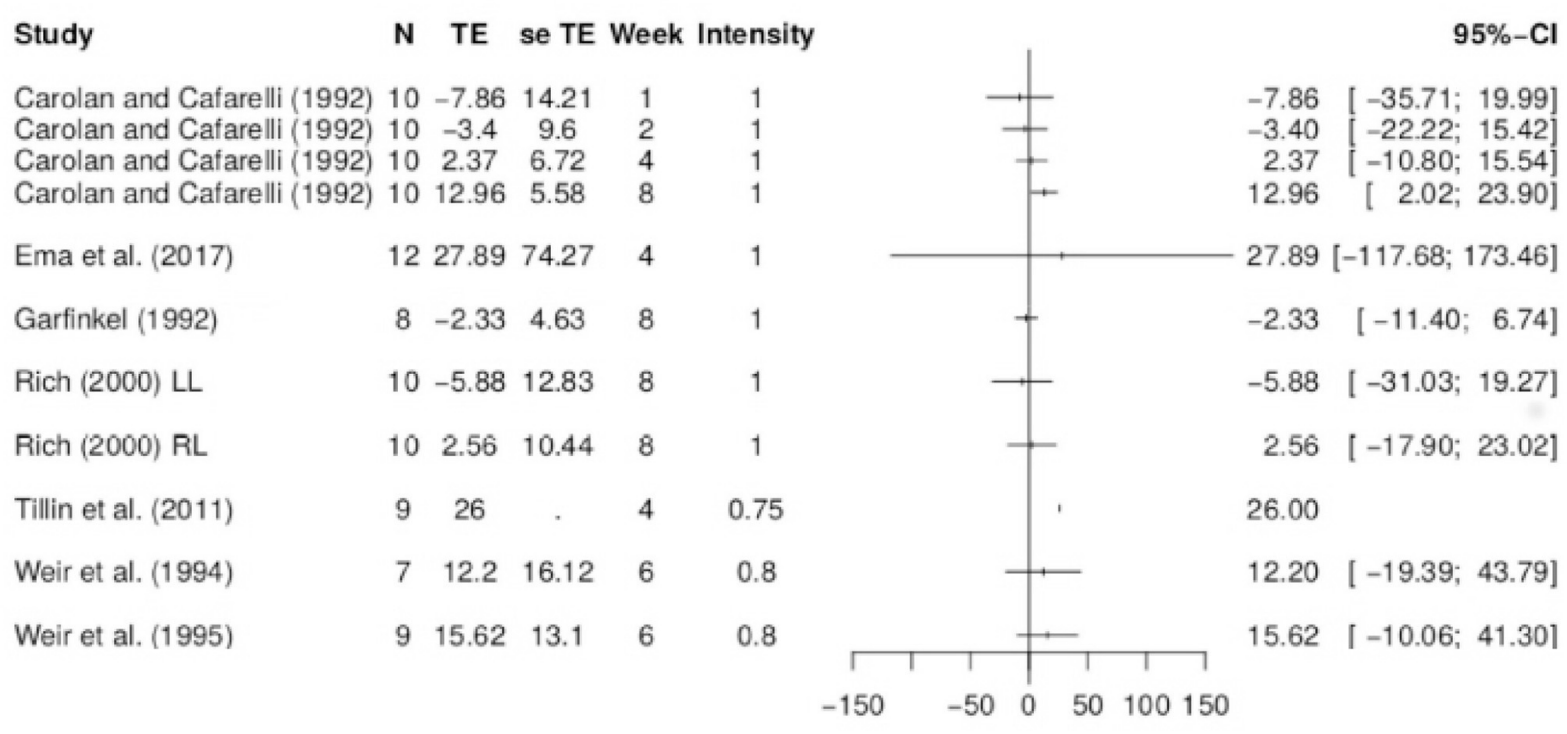
Forest plot for the data on EMG. Here N denotes the number of subjects, TE and se TE denote the mean and standard deviation of the treatment effect, week denotes the time point and intensity denotes the training intensity. A training intensity of 1 corresponds to 100% MVC.

Out of the 20 studies that fulfilled the inclusion criteria, Carolan and Cafarelli (1992), Garfinkel and Cafarelli (1992), Weir et al. (1994, 1995), Rich and Cafarelli (2000), and Ema et al. (2017), reported at 11 data points the EMG values for the vastus lateralis muscle (see Figure 2). Weir et al. (1995) and Ema et al. (2017) additionally reported data on the vastus medialis and rectus femoris. Note that data on the vastus medialis and rectus femoris were excluded from the analysis in order to keep the heterogeneity to a minimum. The relative change in EMG ranges from −7.86 to 27.89% with an average of 7.28 ± 12.48% (see Figure 5).

**Figure 6:**
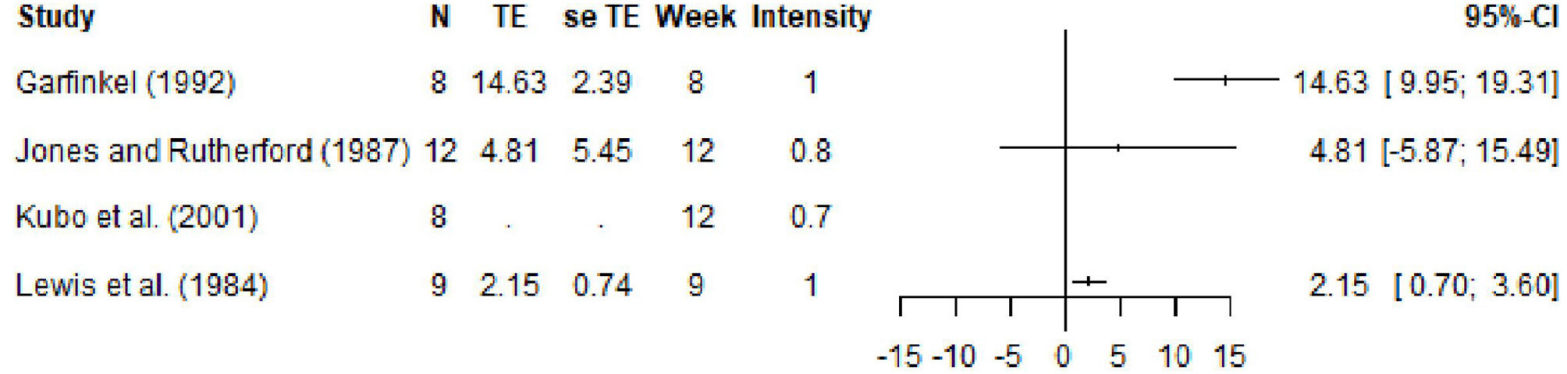
Forest plot for the data on volumetric changes. Here N denotes the number of subjects, TE and se TE denote the mean and standard deviation of the treatment effect, week denotes the time point and intensity denotes the training intensity. A training intensity of 1 corresponds to 100% MVC.

As far as the increase in CSA is concerned, Lewis et al. (1984), Jones and Rutherford (1987) and Garfinkel and Cafarelli (1992) reported an increase of the CSA between 5 and 14.6%, while Kubo et al. (2001) reported an increase of 7.6 ± 4.6% in muscle volume (see Figure 6).

The number of subjects in the trained group across all studies was 307. A total of 163 subjects (54%) were female. Although this study looks like gender balanced (163 female and 144 male subjects have been included), the majority of the female subjects stem from the study of Bandy and Hanten (1993). They report on the outcome of 107 female subjects. If the number of female subjects used in this study is subtracted from the total number of females, only 56 female subjects are left. The age, weight and height of the trained subjects were 22.6 ± 3.1 years, 60.6 ± 9.8 kg, 166.3 ± 8.1 m for the female subjects, and 23.9 ± 3.0 years, 65.3 ± 8.3 kg, 176.0 ± 5.2 m for male subjects, respectively (see Supplementary Table 1). The shortest (Mohr et al., 1985) and longest (Rutherford and Jones, 1986) training periods lasted 3 and 19 weeks, respectively. Qualitatively, the highest rate of change of strength over weeks was reported by Szeto et al. (1989).

For studies included in this systematic review, the training regimen was composed of 6 ± 4 s long contractions, 11 ± 13 repetitions per set, 6 ± 6 sets and rest periods between contractions of 12 ± 13 s and between sets of 1 ± 1 min. The frequency employed in the studies were 4 ± 1 training sessions per week and the total duration of the training sessions went for 7 ± 4 weeks. The intensity was on the average 84 ± 29% MVC with the angle of knee positioned at 78 ± 17°, with 0 degrees corresponding to a fully extended leg. In Szeto et al. (1989), three subject groups were trained with same training volume, but at different levels of intensity (25, 50, 100% MVC). Within the meta-analysis, the groups that trained with 50% MVC (Szeto et al., 1989) and 100% MVC (Szeto et al., 1989) were regarded as two separate groups. The group that trained with 25% MVC was disregarded (see section 2.3).

From the included studies, only Parker (1985) report that the daily activity levels of the subjects were higher than those of recreationally active individual. Jones and Rutherford (1987) provided no information on the activity level of the subjects. Since nothing was explicitly stated in this study, we considered the subjects as recreationally active individuals. Therefore, only four subjects, the ones in Parker (1985), were considered as recreationally highly active individuals. Total number of trained subjects in the studies included in this review is 307. Subjects in Parker (1985) corresponds to 1.3% of the total trained subjects.

**Figure 7:**
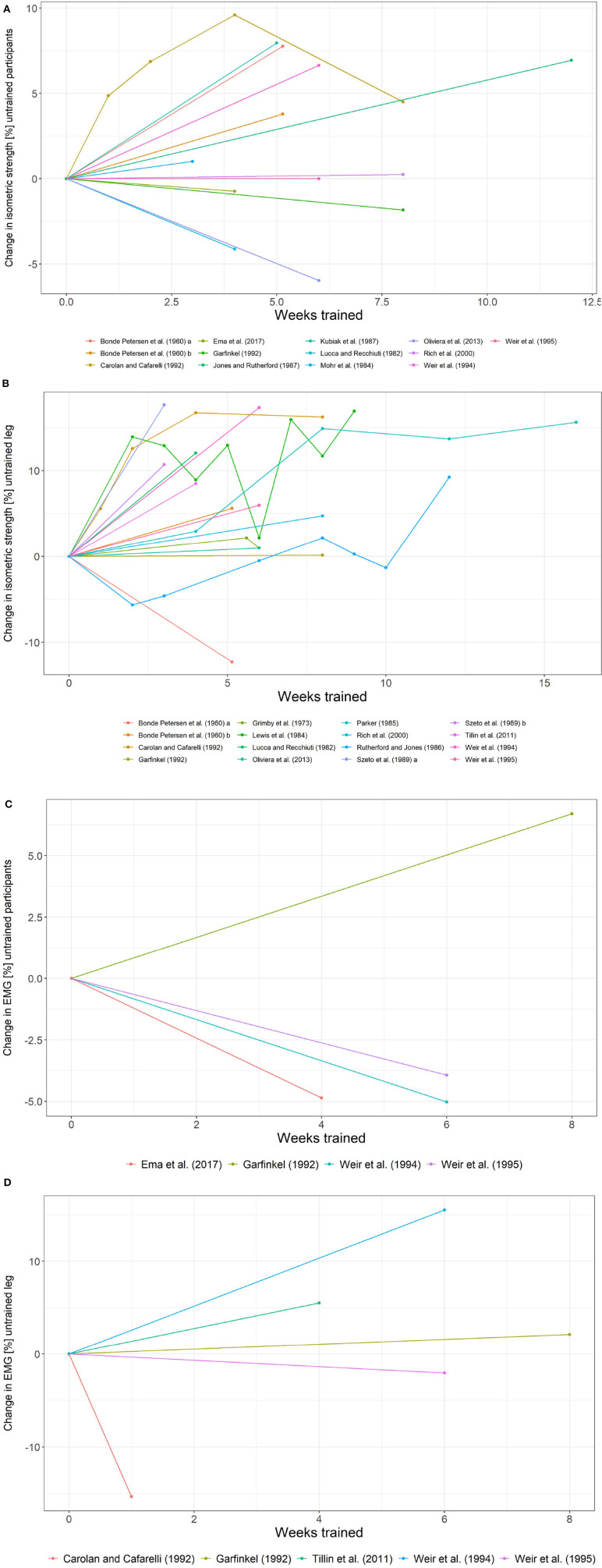
Change in isometric strength and EMG for the control group and untrained leg data. **(A)** Control group data for change in isometric strength. **(B)** Untrained leg data for change in isometric strength. **(C)** Control group data for change in EMG. **(D)** Untrained leg data for change in EMG.

**Figure 8:**
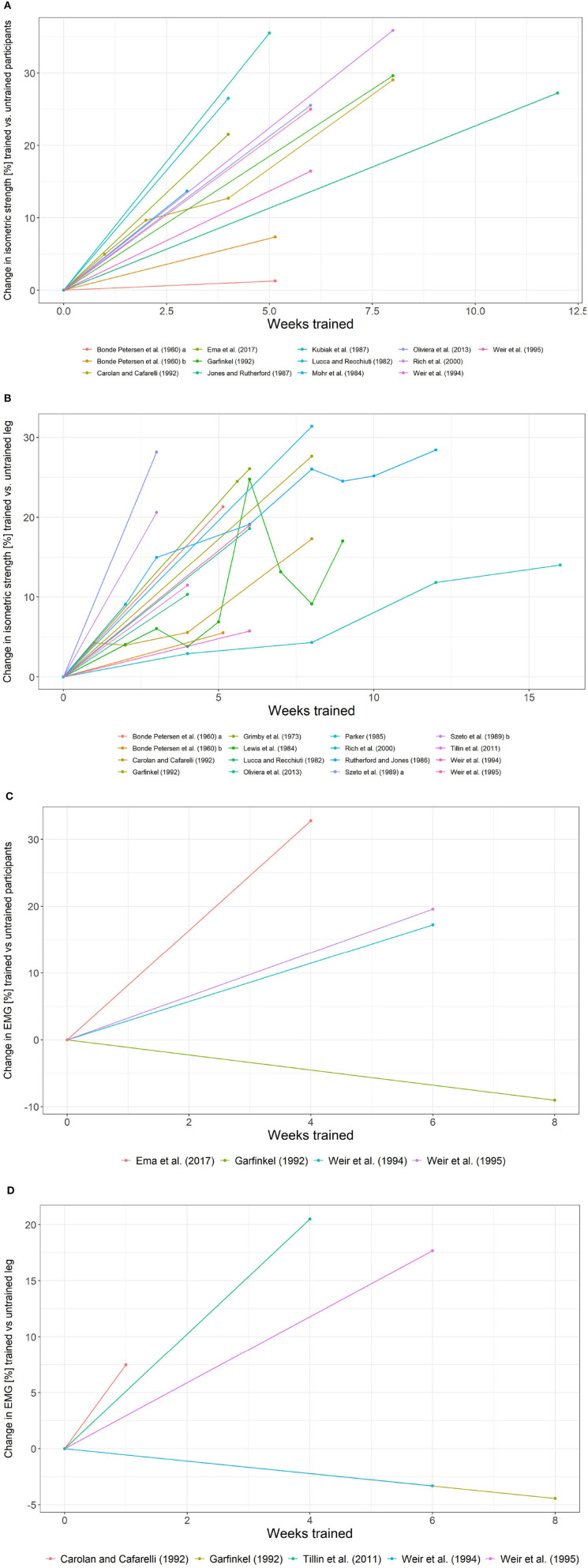
Difference between the change in isometric strength and EMG for the control group and untrained leg data. **(A)** Difference between the change in isometric strength for the trained leg and the control group. **(B)** Difference between the change in isometric strength for the trained leg and the untrained leg. **(C)** Difference between the change in EMG for the trained leg and the control group. **(D)** Difference between the change in EMG for the trained leg and the untrained leg.

As far as control group data are concerned, Bonde Petersen (1960), Lucca and Recchiuti (1983), Carolan and Cafarelli (1992), Garfinkel and Cafarelli (1992), Weir et al. (1994, 1995), and Rich and Cafarelli (2000) report data on a control group and the untrained contralateral leg. Grimby et al. (1973); Bandy and Hanten (1993), and Kubo et al. (2001) do not report any form of control data. Table 1 summarizes the type of control. Spaghetti plots for the control group and untrained leg data are provided in Figure 7. Figure 8 depicts the difference between the change in strength and EMG of the trained leg vs. the control group/untrained leg.

**Table 1:** Breakdown of studies included in the review and if they report data on a control group or the untrained leg (internal control).

**Study**	**Control group**	**Internal control**
Bandy and Hanten (1993)	No	No
Bonde Petersen (1960)	Yes	Yes
Carolan and Cafarelli (1992)	Yes	Yes
Ema et al. (2017)	Yes	No
Garfinkel and Cafarelli (1992)	Yes	Yes
Grimby et al. (1973)	No	No
Jones and Rutherford (1987)	No	Yes
Kubiak et al. (1987)	Yes	No
Kubo et al. (2001)	No	No
Lewis et al. (1984)	No	Yes
Lucca and Recchiuti (1983)	Yes	Yes
Mohr et al. (1985)	Yes	No
Oliveira et al. (2013)	Yes	No
Parker (1985)	No	Yes
Rich and Cafarelli (2000)	Yes	Yes
Rutherford and Jones (1986)	No	Yes
Szeto et al. (1989)	No	Yes
Tillin et al. (2011)	No	Yes
Weir et al. (1994)	Yes	Yes
Weir et al. (1995)	Yes	Yes

*Apart from Grimby et al. (1973), Bandy and Hanten (1993), and Kubo et al. (2001) all studies report data for a control group, an internal control or both*.

The trend of an increase in strength plateauing after week 4 is still prominent for the change in strength when the data on the untrained leg were considered.

The change in isometric strength of the control groups ranges from −8 to 10%, whereas the corresponding change with respect to the untrained leg ranges from −12 to 18%. When the available control group data on the change in strength is taken into consideration (change in strength in the control group subtracted from the change in strength in the trained leg), the change in strength in the trained leg ranges from 1.28 to 35.88%. When data on the untrained leg is considered analogously, the change in strength in the trained leg ranges from 3.96 to 31.38%.

As for the control group data obtained for the vastus lateralis is concerned, the EMG data shows a change between −9.15 and 6.70%, whereas, EMG data for the untrained leg ranges from −15.30 to 15.51%. The difference between the change in EMG for trained leg and the untrained leg is between −9.03 and 19.56%. If the control group is considered, then the difference is −9.03–32.75%. Note, however, that only four data points for EMG data exist for the control group.

### 3.2. Assessment of the Risk of Bias Across Studies

Parker (1985), Szeto et al. (1989), Carolan and Cafarelli (1992), Garfinkel and Cafarelli (1992), and Rich and Cafarelli (2000) stated that the subjects were assigned to training or control groups in a randomized manner. Grimby et al. (1973), Lewis et al. (1984), Parker (1985), Jones and Rutherford (1987), Szeto et al. (1989), Bandy and Hanten (1993), and Tillin et al. (2011) do not employ an untrained control group (see Table 1) and hence do not report on randomization. However, the contralateral leg was considered to be untrained for all those studies since the training mode was unilateral in all studies. Therefore, the fact if a study included a randomized control or not is less significant for the analysis in this study and would not introduce extra heterogeneity to the data.

Food intake and daily activities during the exercising period and previous training experience might effect the strength outcome (Ahtiainen et al., 2016; Damas et al., 2017). Parker (1985), Rutherford and Jones (1986), Bandy and Hanten (1993), and Weir et al. (1995) explicitly state that the participants were instructed to keep these unchanged. Others did not provide explicit information with respect to this concern. Although the study by Garfinkel and Cafarelli (1992) provides no explicit information regarding food intake or daily activity, measuring the mean weight of the subjects before and after the training period revealed that the weight remained unchanged.

In all studies, training took place in a laboratory setting, supervised by one or more researcher. Withdrawal of some subjects due to health reasons or not complying with the exercise protocol occurred in the studies by Bandy and Hanten (1993) and Parker (1985). In these studies, it was stated that the withdrawals did not effect the statistical sensitivity negatively, so the effect of the attrition bias can be regarded as insignificant.

Negative change in strength was only reported in the study of Bonde Petersen (1960). Since the aim of resistance training is to increase muscle strength, negative changes in strength point to over-training or problems with the experimental set-up or the outcome measurement (Fry et al., 1994).

### 3.3. Longitudinal Model-Based Meta-Analysis

BLMBMA allowed us to fit the increase in strength with respect to the baseline strength over time to an Emax model. Since the percent change in strength is modeled, *E*_0_ is taken as 0. The combined maximal increase, which is described by *E*_max_ in Equation (4), was found to be 41.83% (36.60–47.73%) (see Figure 4). Half of the maximal strength increase is reached after 4.39 (3.31–5.84) weeks of training. This time instance described parameter *C*_50_ in Equation (4).

The initial increase in the isometric strength was slightly steeper and becomes flatter over the number of weeks training is performed. This indicates a faster rate of adaptation at the early phases of the exercise period. As some of the individual trials identified by the systematic review were small in size, the shape of the combined curve was heavily influenced by the largest identified study, which estimated the affect most precisely and therefore had a large weight in the meta-analysis. This was also illustrated by the size of the points in Figure 4.

BLMBMA was not feasible for the secondary summary measures (EMG and CSA), since the number of trials was inadequate (7 and 4 studies with 13 and 4 data points, respectively). Therefore, one can only include these data in the analysis according to the last available time point.

**Figure 9:**
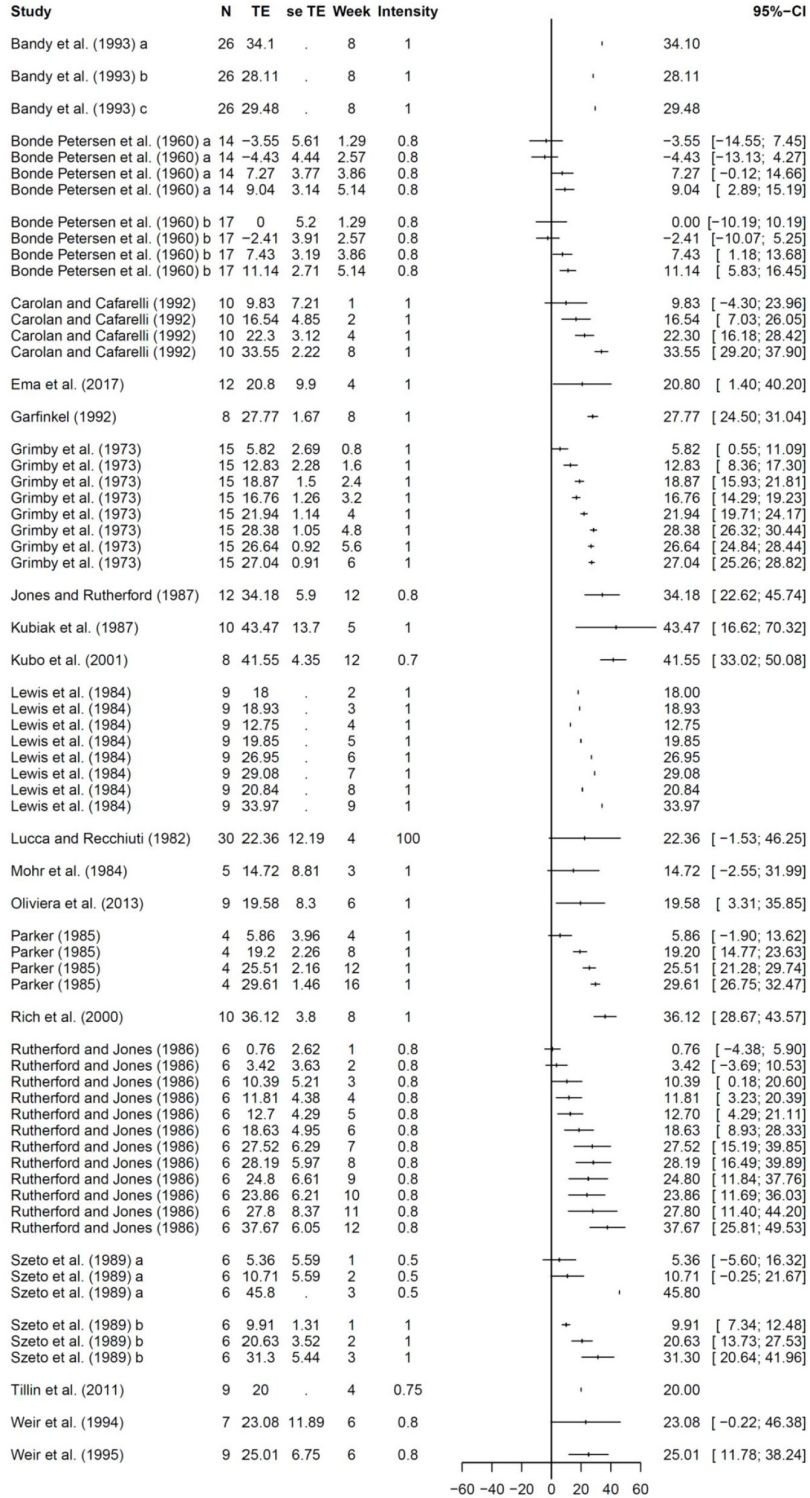
Forest plot of the data on change in isometric strength. Here N denotes the number of subjects, TE and se TE denote the mean and standard deviation of the treatment effect, week denotes the time point and intensity denotes the training intensity. A training intensity of 1 corresponds to 100% MVC.

The landmark analysis of all three outcomes indicate considerable variation between the different trials and time points, e.g., with point estimates that varied in the 65 time points using different follow-up lengths between −4.43 and 43.47 (see Figure 9). Three additional explanatory covariates, namely the proportion of males, mean age and intensity were included. These variables did not explain the observed heterogeneity.

## 4. Discussion

### 4.1. Summary of the Major Findings

This study aims to provide an overview of the UIKEE training effect in healthy, sedentary subjects. The systematic review yielded 20 studies, which fulfilled the inclusion and exclusion criteria. The time-course of the main treatment effect, i.e., the change in isometric strength, could be modeled using a BLMBMA and Emax model. This model showed that isometric strength can increase up to 46%. Half of the increase occurs after the fourth (4.39 ± 1.45 weeks) week of training. The treatment effect of the secondary summary measures, namely the change in cross-sectional area and EMG, could not be modeled longitudinally using BLMBMA, since the number of data points was insufficient due to lack of data.

Pooling data gathered from different studies in form of a systematic review yields a data set with a sample size larger than the subject space of a single experimental study. Further, a longitudinal model-based meta-analysis allows to investigate the time-dependent effects of a given intervention from multiple studies, with different duration and data at different time points. The longitudinal model-based meta-analysis demonstrated its suitability for training data obtained from a quantitative description of the time-dependent training effects of the respective muscles.

### 4.2. Parameters That Influence the Primary Summary Measure

Gender (Donnelly and Smith, 2005; Ribeiro et al., 2014a,b) and the overall general physical condition of the subjects play a (significant) role (Ritti-Dias et al., 2011; Hrysomallis and Buttifant, 2012; Benton et al., 2013; Ribeiro et al., 2014b; do Nascimento et al., 2017; Weakley et al., 2017). The overall number of subjects (see section 3.1) are such that one would not expect much impact. Since there is only one study that reported on subjects with daily activity levels higher than those of recreationally active individuals (Parker, 1985) and one study that did not provide any information about this (Jones and Rutherford, 1987), we believe that the activity of the subjects might be comparable. Hence, we believe that the physical condition, i.e., if non-active or athletes perform the same isometric training program (Patrick and Caterisano, 2002), did not influence the outcome of this meta analysis.

A further key parameter is the training protocol and the time period for which the training was maintained, i.e., the number of trained weeks. The meta-analysis studies investigating the effects of exercise on strength consider the number of weeks trained as an independent variable (e.g., Munn et al., 2004). Lesinski et al. (2016) investigated the effect of various resistance training protocols on the observed changes based on a dose-response relationship, such as training frequency and training intensity. Apart from investigating the effects of individual training variables on the response, a more generic measure describing the “amount” of exercise a muscle was exposed over the course of training, could be established. Such an “amount” could be then associated with the training volume that could be regarded as a specialized time measure depicting the cumulative training volume.

### 4.3. Secondary Summary Measures

Resistance training is one type of training that leads to muscle hypertrophy (see e.g., Wernbom et al., 2007). Hence, the amount and type of training should have a direct impact on muscle hypertrophy. Although isometric knee extension training is one type of resistance training, only 4 out of 20 studies quantified hypertrophy. In these studies, all data showed an increase in either the CSA or the volume of the knee extensors. This is, for example, consistent with Oranchuk et al. (2019). The low number of reported data on hypertrophy is unfortunate, since additional experimental data on hypertrophy would provide a much better insight into the influences of UIKEE induced volumetric muscle growth. However, the available data in Lewis et al. (1984), Jones and Rutherford (1987), Garfinkel and Cafarelli (1992), and Kubo et al. (2001) are insufficient for a quantitative analysis.

Besides pure anatomical measures, i.e., muscle hypertrophy, training has an effect on the functional (physiological) aspects of skeletal muscles, e.g., on neural adaptation (Jones and Rutherford, 1987; Griffin and Cafarelli, 2005; Wernbom et al., 2007). One way of identifying neural adaptation is by recording EMG (Felici, 2006). For example, the change in integrated EMG or the root mean square of the recorded signal are common ways of investigating the extent of neural adaptation due to exercise (Griffin and Cafarelli, 2005).

Only studies of Carolan and Cafarelli (1992), Garfinkel and Cafarelli (1992), Weir et al. (1994, 1995), Rich and Cafarelli (2000), Tillin et al. (2011), and Ema et al. (2017) reported on EMG data. All of these studies obtained the EMG data from the vastus lateralis. Weir et al. (1995) and Ema et al. (2017) report additionally data from vastus medialis and rectus femoris, respectively, however, since there is only one data point available for these muscles, they were not considered in the analyses. As a result, only 11 data points remained for the EMG data.

Among studies that report EMG data, it is only Carolan and Cafarelli (1992), who measured EMG data for multiple time instances, i.e., at 1, 2, 4, and 8 weeks. The rest of the studies reported data only for the end of the training period. Given the fact that the duration of training differed between the studies, the time instances at which the data was recorded and reported also scatter. As a consequence, BLMBMA could not be performed on the EMG data. Despite being informative, EMG data also might be a less reliable source of data for identifying the functional adaptations of training (Arabadzhiev et al., 2014). For example, estimating motor units from EMG is a biased process favoring motor units closer to the surface (Farina et al., 2010).

To significantly improve the information content of the collected UIKEE data would require further measurements, such as, for example, voluntary activation, antagonist coactivation and the rate of force development. Without such data, it is difficult to judge neural adaptation in response to isometric exercise. Among studies that report data on EMG, only some made additional measurements to investigate neural adaptation. Carolan and Cafarelli (1992) and Tillin et al. (2011) reported that hamstring coactivation during UIKEE decreased significantly, which is attributed to neural adaptation mechanisms. Tillin et al. (2011) and Ema et al. (2017) report a significant increase in voluntary activation of the knee extensors (from 89.4 ± 7.0% to 92.5 ± 6.4%) correlated with the relative change in knee extensor isometric strength, whereas, Tillin et al. (2011) did not find any changes in the voluntary activation. Carolan and Cafarelli (1992), Garfinkel and Cafarelli (1992), and Rich and Cafarelli (2000) reported no change in the EMG amplitude. Weir et al. (1994, 1995) detected changes in the EMG amplitude, which were, however, not significant. Rich and Cafarelli (2000) investigated changes in the motor unit firing rate of vastus lateralis, but they did not detect any changes. Note that EMG data was selected as a secondary summary measure. Note that studies, which do investigate neural adaptation, might have discarded as they do not report on muscle strength, which was the inclusion criteria.

### 4.4. Availability of Control Data and Its Use in This Study

The BLMBMA could only be performed for data originating from the trained leg. There existed only five studies that reported data on a control group and the untrained leg. Furthermore, only Carolan and Cafarelli (1992) reported on intermediate data points. Considering the control group and untrained leg data would increase the accuracy of the analysis, however, this would decrease the size of the already sparse data set even further. Moreover, the difference between the treatment effect of the untrained leg and the control group would make it possible to comment on the contralateral training effect, however, data are too sparse to reach a conclusion on this matter. Further, data on the untrained leg is subject-dependent, whereas control group data is a result of an independent observation. The subject dependency cannot be taken into consideration as the mean difference between the data sets is not reported. Therefore, data on the untrained and trained legs cannot be combined.

### 4.5. Comparison of the Findings With Similar Studies

In Oranchuk et al. (2019), the authors focused on the longitudinal adaptation due to isometric training and investigated changes in the morphological, neurological and performance-related properties. They did not distinguish between unilateral and bilateral training and pooled data from studies on large muscles. Furthermore, they included studies training programs that use exercises other than isometric exercise, such as counter-movement jumps. Bohm et al. (2015) investigated the effects of dynamic and isometric exercise on the stiffness, Young's modulus and cross-sectional area of tendons. They found that all exercise modes (eccentric, concentric, and isometric) significantly increased these properties when performed with high intensity (for isometric exercise, more than 70% MVC) and became more significant with increased training duration (more than 8 weeks, up to 3 months). In this study, we disregarded the effect of UIKEE on tendon tissue, however, the fact that Bohm et al. (2015) showed that high intensity training triggers adaptation in tendon tissue, in a way, supports the fact that moderate to high training intensity is required to observe improvements to the musculoskeletal systems.

#### Comparison of the Findings With Bilateral Training Studies

Bilateral and unilateral isometric training are both known to improve muscle function. However, the magnitude of the increase in isometric strength, is known to be less for bilateral training due to the bilateral deficit phenomenon (Howard and Enoka, 1991). Here, a comparison of the effect of bilateral training on the isometric strength with the findings of our study is provided.

Maffiuletti and Martin (2001) studies bilateral isometric knee extension training. They trained two subject groups with ballistic and progressive contractions over 7 weeks and observed 15.7 and 27.4% improvement in isometric strength at the trained angle, respectively. At 7 weeks, our model predicts a 26% increase in isometric strength. In Kubo et al. (2009), subjects were trained with isometric and dynamic knee extensions on either side of the limb. Despite the fact that different training modes were used for each limb, this study can also be regarded as a bilateral training study as both limbs were trained simultaneously. After 14 weeks of training, isometric training yielded 49% increase in isometric strength, whereas, dynamic training yielded 32% increase in isometric strength, i.e., MVC. Neural and morphological adaptation were found to be equal for both sides, where isometric training caused within the isometrically trained leg a 4.5% increase in the cross-sectional area. At 14 weeks of training, our model predicts up to 46% increase in the isometric strength. Similar to their previous studies (Kubo et al., 2001, 2006a,b, 2007), they further found that isometric training is more effective than dynamic training in improving the tendon stiffness and cross-sectional area. It is known that dynamic training also increases isometric strength (e.g., Kanehisa and Miyashita, 1983), thus the greater improvement in isometric strength in Kubo et al. (2009) compared to our findings, may be attributed to the cross-training effect that yields from dynamic training of the contralateral side.

### 4.6. The Advantage of BLMBMA for Computational Modeling

A further advantage of BLMBMA is the availability of a larger and more comprehensive sets of data for computational modeling. Computational models have on one side the ability to systematically analyse complex mechanism and to augment data sets that cannot be recorded for ethical or technical reasons, but also need data for parameter calibration, verification, and validation. This requires a comprehensive and sizeable set of data. Detailed computational models that have been calibrated and validated can help to gain a much better and deeper understanding of the musculoskeletal system. The ability to increase the data set by pooling and merging increase the available data for parameter estimation, verification and validation, and therefore increase the predictability of computational models.

### 4.7. Limitations

Like any study, this study has its limitations. To begin with, experimental studies on muscle adaptation are mainly cohort trials without a control group. If a control group is considered at all, they use an internal control, i.e., the untrained leg (e.g., Jones and Rutherford, 1987). However, it is known that cohort studies are more prone to bias than randomized control trials (Higgins and Green, 2008). For example, if one computes the treatment effect of the trained leg based on the isometric strength of the untrained leg, the computed change in strength would include the cross-training effect. Taking the control as the baseline strength of the trained leg, one would obtain an unbiased way to compute the treatment effect.

Another aspect is that model-based meta-analysis method provides point estimates for the most likely Emax and *C*_50_ parameters and their respective confidence intervals. As beneficial simultaneous confidence intervals and confidence bands for the fitted curve would be, there exists no method that provides those simultaneously. This, however, would be beneficial since confidence bands represent the precision of the estimated curve rather than estimating confidence of individual parameters. Moreover, the between-trial heterogeneity is included by setting a random-effect on the Emax parameter. While this is directly interpretable, it is difficult to directly compare this random-effect to the ones from the landmark analyses heterogeneity measures, as the *I*^2^ does not apply any more. This is due to the fact that variances of the between-trial heterogeneities for the different models relate to different parameters. To elaborate, the *E*_max_ parameter is expected to vary less than the mean effect in the landmark analysis at a same amount of heterogeneity.

Some limitations associated with the current systematic review also include possible publication bias. If there exist studies, which remained unpublished since they found statistically insignificant findings or data were reported in an unpublished dissertation, i.e., gray literature (e.g., Borenstein et al., 2011), it is difficult to spot them by means of electronic search engines. Furthermore, we only included studies published in English and did not investigate studies published in other languages. However, to evaluate the quality and findings of a study which is not written in a language known to the authors, who performed the review, would be difficult. One other limitation regarding the current study is the lack of prospective registration. The systematic review was, nevertheless, conducted according to the remaining items of the PRISMA guideline.

Training intensity is important for initiating improvements in muscle performance (Kraemer and Ratamess, 2004). In this study, we only considered reported data with a minimum of 50% MVC isometric training intensity (c.f., Munn et al., 2004). One could argue that this poses a potential loss of data and effects our interpretation of the change in isometric strength. However, it is known that training protocols with moderate to high intensity trigger hormonal changes that would induce improved muscle performance (c.f., Kraemer and Ratamess, 2004), while training protocols with low intensity isometric exercise mostly effect blood pressure levels (c.f., Taylor et al., 2003; Owen et al., 2010; Carlson et al., 2014; Inder et al., 2016). Among the studies included in this review, only Szeto et al. (1989) reported also on the outcome of a low intensity training group, i.e., a group that trained at 25% MVC. Despite reporting on an increase in isometric strength, the increase was not statistically significant. Therefore one can conclude that the selected level of training intensity matches the intensity required to trigger an improvement in muscle performance.

Furthermore, the reported MVC might be a source for discussion. Lewis et al. (1984) employed 60 maximum isometric contractions in a single training session, in total 120 repetitions in two session in 1 day. This is the highest number of repetitions among the 20 studies fulfilling the inclusion/exclusion criteria of our study. The authors of the study report that despite encouraging subjects to exert maximal effort, the force output corresponded after 3–4 to 70% MVC repetitions and remained at this level for the rest of the session. This finding shows that maximal effort probably cannot be attained consistently for all repetitions in maximal effort training programs. It is therefore important to consider acute fatigue when evaluating the outcomes of maximal effort training.

### 4.8. Outlook

Utilizing a longitudinal model-based meta-analysis will provide new insights into time-dependent effects of a given intervention based on data from multiple studies, with different duration and data at different intervals. One of the biggest profiteers are computational models. The large pool of longitudinal data allows for improved model parameter estimations and provide (sufficient) data for model validation.

Using models to reveal the underlying principles of training do not necessarily exist on the musculoskeletal system level yet. However, such models can be integrated into multi-muscle forward-dynamics models (e.g., Röhrle et al., 2017; Valentin et al., 2018), in neuromuscular system models (e.g., Röhrle et al., 2019) and linked to homogenization techniques aiming to describe macroscopic muscle behavior based on microscopic constituents (e.g., Bleiler et al., 2019). Such models can then be used to investigate the musculoskeletal system in more detail. Further, the data can be used to enhance, integrate and tune detailed biophysical skeletal muscle models (e.g., Heidlauf and Röhrle, 2014) to investigate how (isometric) training would alter active resistance to joint perturbations and therefore the electromechanical delay (e.g., Schmid et al., 2019). These computational models, together with an EMG model, e.g., the one proposed by Klotz et al. (2019), would allow us to better interpret how experimentally determined EMG signals can be linked to changes in cellular processes and force production.

## 5. Summary and Conclusions

This study aims to provide an overview of the training effect of UIKEE performed by healthy, sedentary subjects. The systematic review yielded 20 publications fulfilling the inclusion and exclusion criteria. The time-course of the main treatment effect, i.e., the change in isometric strength, could be modeled using BLMBMA by using the Emax model. The model predicts that the isometric strength can increase up to 46%, where half of the increase occurred at the fourth week of training. It was not possible to model the treatment effect of the secondary summary measures (change in CSA and EMG) longitudinally using BLMBMA due to insufficient data. If the number of controlled trials consistently reporting data on both a separate control group and the untrained leg would have been available, which also take the subject-dependency into consideration, a significant improvement for the analysis of the training effect would have been achieved. In the case of our study, to keep size of the data set at a maximum, all available data on the trained leg were used even if a control group was not reported. The interpretation of the training effect was only possible based on the change from the baseline values, which also allowed to disregard differences in measurement methods e.g., measurement of strength through force or torque output.

## Data Availability Statement

The raw data supporting the conclusions of this article will be made available by the authors, without undue reservation, to any qualified researcher. Requests to access the datasets should be directed to Ekin Altan, altan@imsb.uni-stuttgart.de.

## Author Contributions

EA designed the study, performed the systematic review, extracted the data, assessed the risk of bias among studies, wrote the original draft of the manuscript, and finalized it according to feedback provided by other authors. SS assisted in the study design, performed the statistical analyses, wrote the analyses related sections of the manuscript, and edited it. IB performed the systematic review, extracted the data, assessed the risk of bias among studies, and edited the manuscript. LG supervised the study design and edited the manuscript. HE assisted in the study design and edited the manuscript. OR assisted in the study design, contributed to the outline of the manuscript, partially wrote, and edited the manuscript. All authors contributed to the article and approved the submitted version.

## Conflict of Interest

The authors declare that the research was conducted in the absence of any commercial or financial relationships that could be construed as a potential conflict of interest.
