# Supplementary material for: A Systematic Review and Meta-Analysis on the Longitudinal Effects of Unilateral Knee Extension Exercise on Muscle Strength
Source: Front Sports Act Living. 2020 Nov 16;2:518148. doi: 10.3389/fspor.2020.518148 (PMC7739592; doi:10.3389/fspor.2020.518148)
Supplement: Supplementary file 1 [file Table_1.pdf]

Supplementary Material

1 SEARCH STRATEGY

Keywords, which are used in the review process are categorized as describing

C I the nature of the exercise: ”isometric”,”static”
C II training: ”training”,”exercise”
C III which the body part is being trained: ”knee extensor”,”lower limb”,”quadriceps”
C IV the undesired subject group: ”medicated”,”ill”,”illness”,”sickness”, ”elder”,”elderly”,”old”,”ostheoartritis”

All permutations of each keyword in categories **C I** – **C III** are created and the keywords are combined with an AND. This yields 12 different cases. How each category was combined is illustrated in the following:

1. isometric AND training AND knee extensor
2. static AND training AND knee extensor
3. isometric AND exercise AND knee extensor
4. static AND exercise AND knee extensor
5. isometric AND training AND upper limb
6. static AND training AND upper limb
7. isometric AND exercise AND upper limb
8. static AND exercise AND upper limb
9. isometric AND training AND quadriceps
10. static AND training AND quadriceps
11. isometric AND exercise AND quadriceps
12. static AND exercise AND quadriceps

Keywords in **C IV** were added to each combination with NOT, since papers with these keywords are to be excluded. Note that for the search in Science Direct, only 8 Boolean operators are allowed. Therefore, only ”ill”, ”elder”, ”old”, ”osteoarthritis”, ”handgrip” are added among the keywords in **C IV**.

Below are the search strategies for each database for search case number 1 (see above) in terms of the database’s own syntax:

PubMed:
isometric[Title/Abstract] AND training[Title/Abstract] AND knee extensor[Title/Abstract] NOT medicated[Title/Abstract] NOT ill[Title/Abstract] NOT illness[Title/Abstract] NOT sickness[Title/Abstract] NOT elder[Title/Abstract] NOT elderly[Title/Abstract] NOT old[Title/Abstract] NOT osteoarthritis[Title/Abstract] NOT handgrip

Web of Science:
TI=(isometric) AND TI=(training) AND TI=(knee* AND extensor) NOT TI=(medicated) NOT

TI=(ill) NOT TI=(illness) NOT TI=(sickness) NOT TI=(elder) NOT TI=(elderly) NOT TI=(old) NOT TI=(ostheoartritis) NOT TI=(handgrip)

Science Direct (under the section ”Title, abstract or author-specified keywords” in the advanced search): isometric AND training AND knee extensor NOT ill NOT elder NOT old NOT ostheoastritis NOT handgrip

Chochrane Library:
(isometric):ti,ab,kw AND (training)ti,ab,kw AND (”knee extensor”)ti,ab,kw NOT (medicated)ti,ab,kw NOT (ill)ti,ab,kw NOT (illness)ti,ab,kw NOT (sickness)ti,ab,kw NOT (elder)ti,ab,kw NOT (elderly)ti,ab,kw NOT (old)ti,ab,kw NOT (ostheoartritis)ti,ab,kw NOT (handgrip)

2 RISK OF BIAS ASSESSMENT

The risk of bias (RoB) of individual studies were assessed using the Risk of Bias Tool, version 2 (Sterne et al., 2019). The study design is selected as “individually randomized parallel-group trial”. The outcome of the being assessed is specified as ‘strength’. The reviewer team’s aim for this result is selected as “to assess the effect of adhering to training intervention”. Deviations from intended interventions are selected according to “failures in implementing the intervention that could have affected the outcome”. The crib sheet for the tool is used as a guideline when assessing the RoB of each study (see `https://drive.google.com/file/d/1Q4Fk3HCuBRwIDWTGZa5oH11OdR4Gbhdo/view`). Answers to the questions provided in the tool were given according to the following criteria:

- Bias due to the randomization process: The trained side of the limb (right/left) was assessed by checking if the choice was made based on the dominance of the limb or by random assignment. If the trained side was assigned according to limb dominance, the bias type is selected to raise ”some concerns”, since dominant limbs are known to have a higher baseline strength. Baseline characteristics of the subject groups were assessed according to group size, variation in the demographical information and baseline muscle strength.
- Bias due to deviations from intended interventions: This RoB was assessed based on whether it was reported that participants were instructed to keep the food intake and daily activity unaltered. Furthermore, any manipulations to the trained muscle, such as taking a biopsy from the muscle during the training period, was also assessed. Studies were further investigated, if verbal encouragement or biofeedback was provided during maximal force exertion.
- Bias due to missing outcome data: This bias type was assessed whether the number of subjects that withdrew from the study was statistically significant.
- Bias due to presentation of the measured results: This bias was assessed if results of the intermediary as well as pre-and post-training tests were reported in a tabular form or within a figure.
- Bias due to the selection of the reported result: Studies included in the review were assessed whether they report outcomes of the training effect they investigate and which were described in the methodology.

The outcome of the RoB assessment for each study is given in Table S1.

Table S1. The results of risk of bias assessment for each study. Here, SC denotes some concerns, LR denotes low risk and HR denotes high risk.

Study	Randomization process	Deviations from intended interventions	Missing outcome data	Measurement of the outcome	Selection of the reported result	Overall Bias
Bonde-Petersen (1960)	SC	HR	LR	LR	LR	SC
Carolan and Caferelli (1992)	LR	SC	LR	LR	LR	LR
Ema et al. (2017)	LR	LR	LR	LR	LR	LR
Garfinkel and Caferelli (1992)	LR	LR	LR	LR	LR	LR
Jones and Rutherford (1987)	LR	LR	LR	LR	LR	LR
Kubiak et al. (1987)	LR	SC	LR	LR	LR	LR
Lewis et al. (1984)	LR	LR	LR	LR	LR	HR
Lucca and Recchiuti (1983)	LR	SC	LR	LR	LR	LR
Mohr et al. (1985)	LR	SC	LR	LR	LR	LR
Oliveira et al. (2013)	LR	SC	LR	LR	LR	LR
Parker (1985)	SC	SC	LR	LR	LR	SC
Rich and Cafarelli (2000)	LR	SC	LR	LR	LR	LR
Rutherford and Jones (1986)	LR	LR	LR	LR	LR	LR
Szeto et al. (1989)	LR	SC	LR	LR	LR	LR
Tillin et al. (2011)	LR	SC	LR	LR	LR	LR
Weir et al. (1994)	LR	LR	LR	LR	LR	LR
Weir et al. (1995)	LR	LR	LR	LR	LR	LR

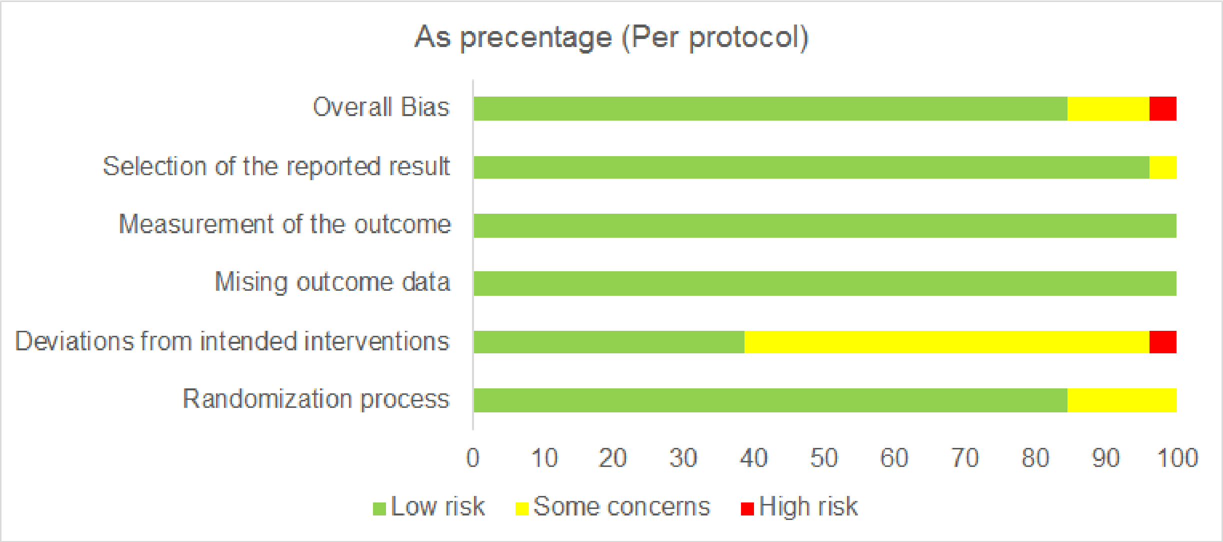

Figure S1. The results of risk of bias assessment using Risk of Bias Tool, version 2. A potentially high RoB is observed in the study by Lewis et al. (1984), since they do not report when the tissue biopsy was obtained.

Table S2. Demographical information on subjects for studies included in the meta-analysis, part I. T: trained group, C: control group, NI: no information, [1]: mean and standard deviation was not provided, only a range of values was given, [2]: mean and standard deviation for male and female subjects were not given separately, [3]: mean and std for training and control group were not given separately. Note that in Kubiak et al. 1987, did not give information on how male and female subjects were distributed among the trained and control groups. The subject numbers for trained/control, female/male were assumptions.

		Female				Male			
**Code**	**#**	**#**	**Age**	**Mass [kg]**	**Height [cm]**	**#**	**Age**	**Weight [kg]**	**Height [cm]**
Bandy and Hanten1993 T	107	107	23.9 ± 4.3	NI	NI	0	0	0	0
Bonde Petersen 1960 T [1,2,3]	31	14	24	NI	NI	17	24	NI	0
Bonde Petersen 1960 C [1,2,3]	13	7	24	NI	NI	6	24	NI	0
Carolan and Cafarelli 1992 T	10	0	0	0	0	10	21.4 ± 0.7	77.7 ± 3.6	176.4 ± 4.7
Carolan and Cafarelli 1992 C	10	0	0	0	0	10	22.1 ± 0.9	76.2 ± 3.2	177.9 ± 4.4
Ema et al. 2017 T	12	0	0	0	0	12	22±3	61±7	170±4
Ema et al. 2017 C	11	0	0	0	0	11	22±4	62±6	170±7
Garfinkel and Cafarelli 1992 T	8	8	NI	NI	NI	0	0	0	0
Garfinkel and Cafarelli 1992 C	7	7	NI	NI	NI	0	0	0	0
Grimby et al. 1973 T [1]	15	0	0	0	0	15	22-32	NI	NI
Jones and Rutherford 1987 T	12	1	NI	NI	NI	11	27.5 ± 5.7	64.5 ± 7.3	171.5 ± 6.4
Kubiak et al. 1987 T [1,2,3]	10	NI	18-30	NI	NI	NI	18-30	NI	NI
Kubiak et al. 1987 C [1,2,3]	9	NI	18-30	NI	NI	NI	18-30	NI	NI
Kubo et al. 2001 T	8	0	0	0	0	8	22.6 ± 2.8	69.2 ± 5.8	171.5 ± 6.1
Lewis et al. 1984 T	9	0	0	0	0	9	23	74	185
Lucca and Recchiuti 1983 T	10	10	20.0 ± 0.2	58.5 ±166.8 8.8	166.8 ± 5.8	0	0	0	0
Lucca and Recchiuti 1983 C	10	10	20.3 ± 0.48	55.4 ± 5.7	165.4 ± 6.7	0	0	0	0
Mohr et al. 1985 T [1,3]	5	5	21-29	NI	NI	0	0	0	0
Mohr et al. 1985 C [1,3]	6	6	21-29	NI	NI	0	0	0	0
Oliveira et al. 2013 T [3]	9	0	0	0	0	9	23.2±3.8	79.1±11.5	178±0.08
Oliveira et al. 2013 C [3]	9	0	0	0	0	9	23.2±3.8	79.1±11.5	178±0.08
Parker 1985 T	4	0	0	0	0	4	26.5 ± 2.6	81.1 ± 8.6	182 ± 4
Rich and Cafarelli 2000 T	10	0	0	0	0	10	NI	NI	NI
Rich and Cafarelli 2000 C	10	0	0	0	0	10	NI	NI	NI
Rutherford and Jones 1986 T	6	0	0	0	0	6	27.50	64.5 ± 7.3	171.50
Szeto et al. 1989 T	6	0	0	0	0	6	24 ± 3	65 ± 7	172 ± 6
Tillin et al. 2011 T	9	0	0	0	0	9	21 ± 1	81 ± 7	182 ± 5
Weir et al. 1994 T [1]	7	4	22 ± 4	67 ± 10	NI	3	22 ± 4	67 ± 10	NI
Weir et al. 1994 C [1]	6	3	24 ± 4	72 ± 9	NI	3	24 ± 4	72 ± 9	NI
Weir et al. 1995 T	9	9	20 ± 1	59 ± 8	168 ± 7	0	0	0	0
Weir et al. 1995 C	8	8	21 ± 1	62 ± 7	169 ± 6	0	0	0	0

Table S3. Training variables given in the studies. CD: contraction duraction [s], R1: rest between each contraction [s], R2: rest between each set [min], TD: duration of the whole training period [weeks], Freq: frequency of training [sessions/week], Rep: repetitions, Int.: training intensity [%MVC], Angle: angle in degrees that the knee was trained, 0° corresponds to the fully extended leg.

Ref	CD	R1	R2	TD	Freq	Sets	Rep/set	Int	Angle
Bandy and Hanten1993 a	6	0	0	8	3	20	1	100	30
Bandy and Hanten1993 b	6	0	0	8	3	20	1	100	60
Bandy and Hanten1993 c	6	0	0	8	3	20	1	100	90
Bonde Petersen 1960	6	20	2	4	3	5	5	80	90
Carolan and Cafarelli 1992	4	3	0	8	3	1	30	100	90
Ema et al. 2017	3	17	2	4	3	4	10	100	90
Garfinkel and Cafarelli 1992	4	25	-	8	3	3	10	100	90
Grimby et al. 1973	5	3	0	6	5	1	30	100	90
Jones and Rutherford 1987	4	2	1	12	3	4	6	100	90
Kubiak et al. 1987	10	50	0	5	3	10	1	100	60
Kubo et al. 2001	20	0	1	12	4	4	1	70	90
Lewis et al. 1984	5	10	NI	9	5	2	60	100	90
Lucca and Recchiuti 1983	6	25	0	4	5	5	1	100	60
Mohr et al. 1985	10	10	0	3	5	1	10	100	60
Oliveira et al. 2013	5	15	2	6	3	3	8	100	75
Parker 1985	2	10	0	19	3	1	10	100	90
Rich and Cafarelli 2000	4	NI	NI	8	3	5	10	100	90
Rutherford and Jones 1986	4	2	1	12	3	4	6	80	90
Szeto et al. 1989 a	5	5	1	3	5	3	10	50	90
Szeto et al. 1989 b	5	5	1	3	5	3	10	100	90
Tillin et al. 2011	3	2	2	4	4	4	10	100	85
Weir et al. 1994	6	30	2	6	3	2	10	80	135
Weir et al. 1995	6	30	2	6	3	2	10	80	135
Mean	6	12	1	7	4	6	11	84	78
St.D.	4	13	1	4	1	6	13	29	17

Table S4. Anatomical changes in the knee extensors. All values except for the week column denote the mean TE and when reported ± standard deviation as percent change from the baseline value. The week column corresponds to at which stage the data is reported. CSA: cross-sectional area, NI: no information.

Study	Week	CSA	$\frac{\text{Force}}{\text{Area}}$	Volume
Garfinkel and Cafarelli 1992	8	14.60	0.00	NI
Jones and Rutherford 1987	12	5 ± 4.6	27.27	NI
Kubo et al. 2001	12	NI	NI	7.6 ± 4.3

PRISMA 2009 Checklist

Section/topic	#	Checklist item	Reported on page #
**TITLE**			
Title	1	Identify the report as a systematic review, meta-analysis, or both.	1
**ABSTRACT**			
Structured summary	2	Provide a structured summary including, as applicable: background; objectives; data sources; study eligibility criteria, participants, and interventions; study appraisal and synthesis methods; results; limitations; conclusions and implications of key findings; systematic review registration number.	1
**INTRODUCTION**			
Rationale	3	Describe the rationale for the review in the context of what is already known.	1-2
Objectives	4	Provide an explicit statement of questions being addressed with reference to participants, interventions, comparisons, outcomes, and study design (PICOS).	2
**METHODS**			
Protocol and registration	5	Indicate if a review protocol exists, if and where it can be accessed (e.g., Web address), and, if available, provide registration information including registration number.	-
Eligibility criteria	6	Specify study characteristics (e.g., PICOS, length of follow-up) and report characteristics (e.g., years considered, language, publication status) used as criteria for eligibility, giving rationale.	3
Information sources	7	Describe all information sources (e.g., databases with dates of coverage, contact with study authors to identify additional studies) in the search and date last searched.	2
Search	8	Present full electronic search strategy for at least one database, including any limits used, such that it could be repeated.	in Suppl. Mat.
Study selection	9	State the process for selecting studies (i.e., screening, eligibility, included in systematic review, and, if applicable, included in the meta-analysis).	2
Data collection process	10	Describe method of data extraction from reports (e.g., piloted forms, independently, in duplicate) and any processes for obtaining and confirming data from investigators.	3-4
Data items	11	List and define all variables for which data were sought (e.g., PICOS, funding sources) and any assumptions and simplifications made.	3-4
Risk of bias in individual studies	12	Describe methods used for assessing risk of bias of individual studies (including specification of whether this was done at the study or outcome level), and how this information is to be used in any data synthesis.	4,6
Summary measures	13	State the principal summary measures (e.g., risk ratio, difference in means).	3
Synthesis of results	14	Describe the methods of handling data and combining results of studies, if done, including measures of consistency (e.g., I^2) for each meta-analysis.	4

Section/topic	#	Checklist item	Reported on page #

PRISMA 2009 Checklist

Risk of bias across studies	15	Specify any assessment of risk of bias that may affect the cumulative evidence (e.g., publication bias, selective reporting within studies).	4,6
Additional analyses	16	Describe methods of additional analyses (e.g., sensitivity or subgroup analyses, meta-regression), if done, indicating which were pre-specified.	4
**RESULTS**			
Study selection	17	Give numbers of studies screened, assessed for eligibility, and included in the review, with reasons for exclusions at each stage, ideally with a flow diagram.	7
Study characteristics	18	For each study, present characteristics for which data were extracted (e.g., study size, PICOS, follow-up period) and provide the citations.	6,7,9,14
Risk of bias within studies	19	Present data on risk of bias of each study and, if available, any outcome level assessment (see item 12).	14, Suppl. Mat
Results of individual studies	20	For all outcomes considered (benefits or harms), present, for each study: (a) simple summary data for each intervention group (b) effect estimates and confidence intervals, ideally with a forest plot.	15
Synthesis of results	21	Present results of each meta-analysis done, including confidence intervals and measures of consistency.	8,9,15
Risk of bias across studies	22	Present results of any assessment of risk of bias across studies (see Item 15).	Suppl. Mat
Additional analysis	23	Give results of additional analyses, if done (e.g., sensitivity or subgroup analyses, meta-regression [see Item 16]).	14
**DISCUSSION**			
Summary of evidence	24	Summarize the main findings including the strength of evidence for each main outcome; consider their relevance to key groups (e.g., healthcare providers, users, and policy makers).	14,16
Limitations	25	Discuss limitations at study and outcome level (e.g., risk of bias), and at review-level (e.g., incomplete retrieval of identified research, reporting bias).	17,18
Conclusions	26	Provide a general interpretation of the results in the context of other evidence, and implications for future research.	18
**FUNDING**			
Funding	27	Describe sources of funding for the systematic review and other support (e.g., supply of data); role of funders for the systematic review.	19

From: Moher D, Liberati A, Tetzlaff J, Altman DG, The PRISMA Group (2009). Preferred Reporting Items for Systematic Reviews and Meta-Analyses: The PRISMA Statement. PLoS Med 6(7): e1000097. doi:10.1371/journal.pmed1000097

For more information, visit: **www.prisma-statement.org**.

Page 2 of 2
